# Nailfold Video Capillaroscopy in Pregnant Women With and Without Cardiovascular Risk Factors

**DOI:** 10.3389/fmed.2022.904373

**Published:** 2022-07-05

**Authors:** Kristof Thevissen, Merve Demir, Jerome Cornette, Wilfried Gyselaers

**Affiliations:** ^1^Department of Rheumatology, Ziekenhuis Oost-Limburg (ZOL), Genk, Belgium; ^2^Faculty of Medicine and Life Sciences, Hasselt University, Diepenbeek, Belgium; ^3^Department of Obstetrics and Fetal Medicine, Erasmus Medical Center, Rotterdam, Netherlands; ^4^Department of Physiology, Hasselt University, Diepenbeek, Belgium; ^5^Department of Gynecology, Ziekenhuis Oost-Limburg (ZOL), Genk, Belgium

**Keywords:** Nailfold video capillaroscopy, microcirculation, pregnancy, pre-eclampsia, hypertension

## Abstract

**Objective:**

To evaluate microvasculature in pregnant women with and without cardiovascular risk factors.

**Design:**

Cross-sectional, observational study.

**Population:**

Women were recruited at the outpatient clinic for high risk prenatal care. Out of a total of 345 women assessed at first and/or second and/or third trimester, 169 women without and 176 with cardiovascular risk factors were included.

**Methods:**

Nailfold video capillaroscopy (NVC) measurements were performed at magnification of 200x at all fingers except thumbs. Images were stored for offline measurement of capillary density (CDe) and capillary diameters (CDi). Maternal anthropometrics, obstetric, and medical history were used for categorization in low and high cardiovascular risk. Comparison between groups and trimesters, with respect to pregnancy outcome, was performed using linear mixed model analysis.

**Results:**

Women with a high risk cardiovascular profile show higher CDe, regardless of pregnancy outcome. CDi drops during pregnancy, with lowest CDi in third trimester in patients with preeclampsia. Capillary bed (CB), as a composite of CDe and CDi, is stable during pregnancy in women with low risk cardiovascular profile. In women with high risk cardiovascular profile, CB drops from the first to the second trimester, regardless of pregnancy outcome. Only in women with pre-eclampsia, the CB is lower in the third trimester as compared to the first trimester.

There is an inverse association between CDe and mean arterial pressure (MAP) in women with high cardiovascular risk and pre-eclampsia.

**Conclusion:**

Microcirculation is altered during the course of pregnancy and microcirculatory behavior is different in patients with low and high cardiovascular risk profile, as well as in patients with preeclampsia.

## Introduction

Pregnancy requires profound adaptation in the circulatory system, in order to guarantee adequate blood supply to the mother and fetus (1). From the 5th week of gestation, peripheral vascular resistance (PVR) lowers and cardiac output rises by increasing heart rate and stroke volume. The state of an underfilled cardiovascular system urges a plasma volume expansion through hormonal changes and an increase in sodium and water reabsorption. Two weeks later, venous compliance begins to increase and this phenomenon persists throughout pregnancy in order to build up a buffer compartment in the splanchnic system. Between 8 and 12 weeks of gestation, morphological changes occur in the heart, which can possibly be explained by the continuous higher venous return and the improvement in cardiac performance. In the second half of the first trimester, retrograde trophoblast invasion into the spiral artery is one of the reasons for increase of uteroplacental vascular compliance (1, 2).

There are only sparse data on microcirculatory changes in pregnancy. The available data differ in used techniques and outcome measures (3). In a prospective study, Antonios et al., were able to show that quantifying structural rarefaction of skin capillaries in pregnancy is a potentially useful clinical marker for the prediction of preeclampsia (4). In most studies, microcirculation is studied in a dynamic way, with recruitment maneuvers (e.g., by applying pressure to congest vessels before measurement). Less is known about static features of microcirculation in pregnancy.

Nailfold video capillaroscopy (NVC) is a method for static evaluation of microcirculation at the nailfolds of the fingers at both hands. In daily practice, this technique is used to diagnose and evaluate systemic sclerosis. There, changing patterns can predict internal organ involvement (5). Only few papers exist on NVC in pregnancy (6, 7).

In this study, we aimed to evaluate structural changes in nailfold microcirculation in pregnancies of women with or without cardiovascular risk factors. Our hypothesis is that trimestral measurements of CDi and CDe are similar in uncomplicated pregnancies, regardless of cardiovascular risk.

## Patients and Methods

### Study Design and Population

A cross-sectional, observational study is conducted in the Fetal Medicine Unit of Ziekenhuis Oost-Limburg (Genk, Belgium) to evaluate the microcirculatory adaptations during pregnancy. This hospital has a combined secondary and tertiary referral function. Approval of the local Committee for Medical Ethics of Ziekenhuis Oost-Limburg (Genk, Belgium) and Hasselt University (Hasselt, Belgium) was obtained before study onset (May 29, 2017—filenr. 17/019U). All participants gave written informed consent. Obstetric and clinical history, including age, length, weight before pregnancy, profession, nationality, marital status, number of previous pregnancies, medication use, smoking history, and history of Raynaud’s phenomenon were recorded, as well as blood pressure (BP) and actual weight measurements.

A total of 345 women were assessed in first and/or second and/or third trimester, categorized in 3 groups: one group with low cardiovascular (CV) risk profile, one group with high CV profile but normal pregnancy outcome and one group with high CV profile who developed preeclampsia (PE). For this study our focus was mainly to describe the differences between low and high cardiovascular risk profile and a normal pregnancy outcome. We included also patients with preeclampsia as outcome, to show that our data in that patient group are in line with previous published data, to strengthen our findings in patients with normal pregnancy outcome. Cardiovascular risk profile was based on their anthropometrics, obstetric and medical history, as specified in Table 1. Low risk women were invited to participate during routine antenatal visits, whereas in the high risk group, NVC was part of the maternal cardiovascular assessment performed for clinical indications in the referral clinic for high risk prenatal care (8).

**TABLE 1:** Baseline characteristics of study population.

	Low risk group Group 1	High risk group Normal outcome Group 2	High risk group Preeclampsia Group 3	Group 1 vs. 2	Group 2 vs. 3	Group 1 vs. 3
**N patients**	169	142	34	NA	NA	NA
**Age (mean; SD)**	30.26; 4.38	31.72; 4.26	30.91; 3.88	**0.004**	0.312	0.424
**Smoking**	21/169 (12.43%)	10/142 (7.04%)	3/34 (8.82%)	0.114	0.721	0.552
**Raynaud**	11/169 (6.51%)	10/142 (7.04%)	3/34 (8.82%)	0.852	0.721	0.627
**BMI (mean; SD) *n* = 159 (normal risk)/139 (high risk)/34 (preeclampsia)**	24.75; 5.08	27.18; 6.32	26.68; 5.91	**0.000**	0.674	0.052
**Length (mean; SD) *n* = 159 (normal risk)/139 (high risk)/34 (preeclampsia)**	165.2; 6.64	166.93; 6.38	166.18; 7.17	**0.022**	0.547	0.440
**Weight (mean; SD) *n* = 169 (normal risk)/141 (high risk)/34 (preeclampsia)**	68.03; 15.14	76.94; 18.06	75.70; 19.47	**0.000**	0.726	**0.011**
**Mean Arterial Pressure (mean; SD) = 161 (normal risk)/142 (high risk)/34 (preeclampsia)**	91.42; 8.95^£^	95.32; 11.44	104.68; 13.21	**0.001**	**0.000**	**0.000**
**Parity**				0.0000^¥^		
**Nulliparous**	61.54%	28.87%	59.38%	-	-	-
**Primiparous/multiparous**	38.46%	71.13%	40.63%	-	-	-
**Gestational age at birth (mean; SD)**	39^+1^.^6^; 12.44 (days)	38^+6^; 22.63 (days)	35; 22.63 (days)	0.058	**0.000**	**0.000**
**Mode of delivery *n* = 169 (normal risk)/135 (high risk)/30 (preeclampsia)**				0.247*		
**Vaginal**	81.55%	74.07%	73.33%	-	-	-
**Section**	18.45%	25.93%	26.67%	-	-	-
**Birth Weight in grams (mean; SD) *n* = 168 (normal risk)/133 (high risk—normal outcome)/29 (high risk—preeclampsia)**	3318.76; 537.87	3258.06; 502.37	2257.69; 934.91	0.318	**0.000**	**0.000**
**Percentile (mean; SD) *n* = 168 (normal risk)/129 (high risk—normal outcome)/29 (high risk—preeclampsia)**	52.12; 29.35	49.20; 26.76	35.64; 30.67	0.378	**0.018**	**0.006**
**Medication (other than antihypertensive)**	8.88%	17.04%	21.88%	**0.033**	0.522	**0.031**
**Medication (antihypertensive)**	0%	5.15%	37.50%	**0.003**	**0.000**	**0.000**
**Reason for evaluation in high prenatal care outpatient clinic**	0/169	142/142 (100%)	34/34 (100%)	NA		
**History of abruption placentae**		2/142		-		
**History of eclampsia/pre-eclampsia**		47/142	6/34	-		
**Pre-eclampsia in current pregnancy**		0/142	14/34	-		
**Underlying systemic disease**		10/142		-		
**Newly discovered hypertension**		27/142	9/34	-		
**History of gestational hypertension**		15/142	1/34	-		
**History of HELLP**		8/142	1/34	-		
**Essential hypertension**		4/142	1/34	-		
**History of intra-uterine growth restriction**		8/142		-		
**Newly discovered intra-uterine growth restriction**		2/142		-		
**Age**		1/142		-		
**History of unexplained intrauterine demise**		1/142		-		
**Profound edema**		1/142		-		
**Thrombophilia**		2/142	2/34	-		
**History of premature rupture of membranes**		3/142		-		
**Screening of high uterine artery resistance**		9/142		-		
**History of premature birth**		2/142		-		

*Vaginal vs. section.

*^¥^Nulliparous vs. primi-/multiparous.*

*^£^Based on 161 assessments.*

*NA, not applicable.*

*For continuous variables mean and standard deviation are given, together with a p-value obtained for a t-test. For categorical variables counts and % are given, with a p-value for a Chi-square test (or Fisher Exact test).*

*Bold values are the significant ones.*

### Methods

For capillary assessments, a hand-held video nailfold capillaroscope (Optilia Mediscope, Vällingby, Sweden) was used, equipped with a 200x magnification high-resolution lens.

**FIGURE 1:**
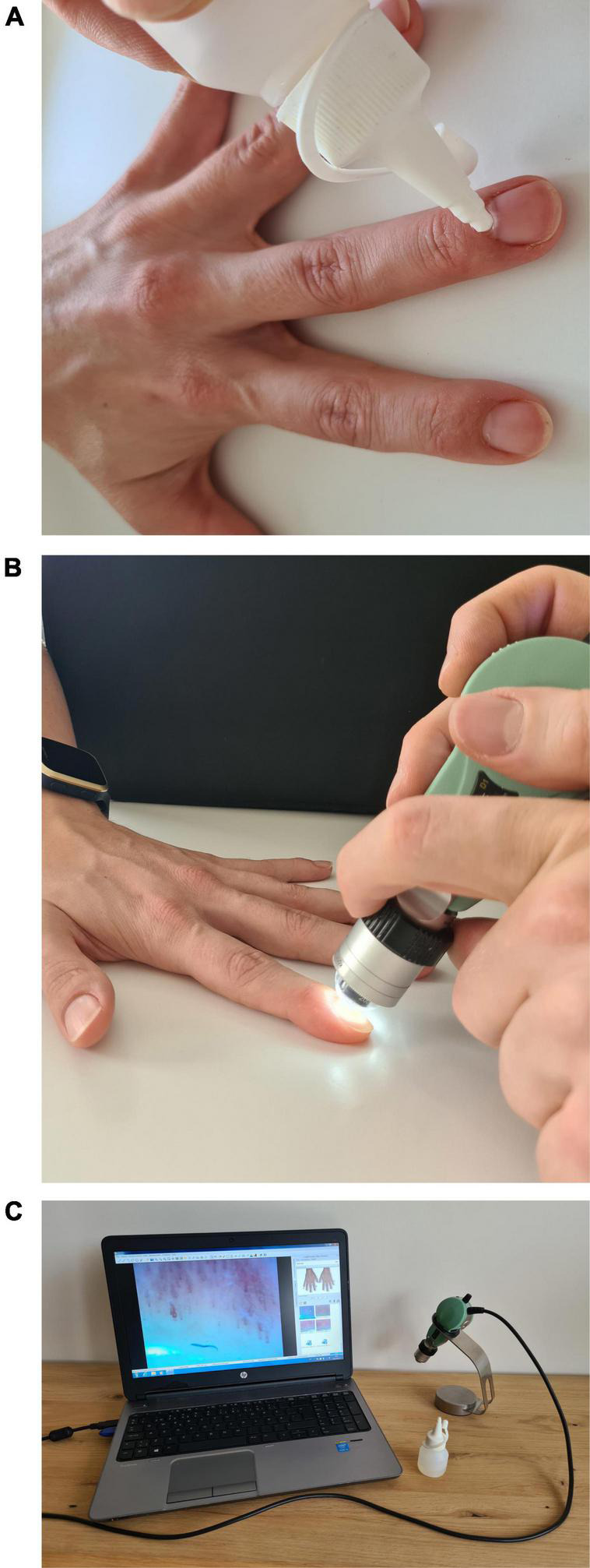
Equipment and performance of NVC. **(A)** Oil application. **(B)** Adjusting. **(C)** Equipment to capture.

**FIGURE 2:**
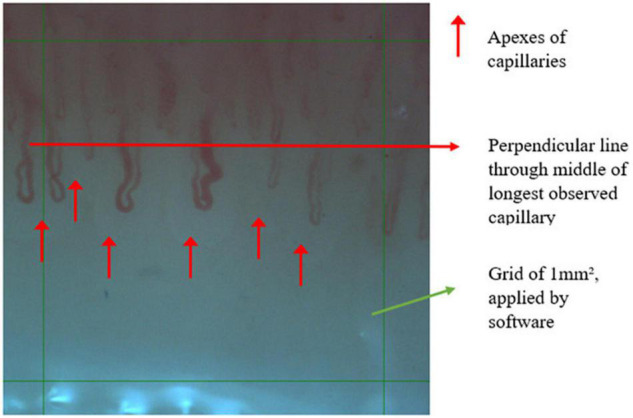
Example of one measured field of 1 mm^2^.

To keep the core temperature of the patient in a normal range to exclude cold induced vasoconstriction, all capillaroscopy measurements were conducted after acclimatization in a temperature-controlled room (22°C), after the obstetric ultrasound scan. Additionally, the equipment and process of measurements were explained to the patient to avoid stress during the measurement (9). Before the NVC examination, patients were seated in a chair with their hands placed at the heart level resting on a table. Next, a small drop of oil was applied on the nailfold of all fingers, except thumbs, to maximize the amount of light and improve the visibility of capillaries. Subsequently, the nailfold of the second, third, fourth, and fifth fingers was examined bilaterally by one researcher (KT) using NVC. In order to reduce selection bias, four consecutive microscopic fields extending over 1 mm centered around the nailfold were studied per finger. The contact angle and the direction of the capillaroscope were manually adjusted to reduce light reflections (9) (see Figure 1). Finally, by manually adapting the focusing system and triggering the button on the device, a sharp image of the capillaries was taken (see Figure 2).

The microscopic images were obtained using the OptiPix system (OptiPix Capillary, version 1.7.7, Optilia, Sweden) and analyzed off-line from the stored data by one observer (KT), blinded to the pregnancy outcome and cardiovascular risk of the patient. The quantitative analysis of NVC changes is a validated technique in systemic sclerosis research, were images are more challenging to capture due to contractures of fingers and thicker nailfolds due to sclerosis (10–13). In earlier research there was a high reproducibility reported for NVC (14). More recent research also uses the technique of NVC in normal pregnancy (15).

CDe is defined as the number of capillaries that are perfused at the time of examination. The analysis of capillaries was performed within the region of interest (ROI), which is a grid of 1 mm^2^, containing the capillaries to be counted and measured. Therefore, every grid is positioned randomly before starting the analysis. All capillary apexes within the ROI were counted manually in two of the four microscopic fields chosen by the observer. The researcher directly observed the loops and indicated the loops that were considered to be distal, as per definition this is distal to a perpendicular line through the middle of longest observed capillary. If the common branch of the capillary positioned within the ROI, it was counted as being one capillary. Additionally, if only the apex of the capillary is present within the ROI, it was also counted as being one capillary.

The CDi was measured as the largest diameter of the erythrocyte column from the apex of the loop, which is also defined as the transitional diameter of a capillary. All quantifiable capillary loops were measured.

We estimated an individual’s total capillary bed surface (CB) by multiplying for each finger the mean CDe with the mean CDi, followed by calculating the mean digital CB per patient by summation of all digital values divided by 8.

Besides nailfold capillaroscopy we also measured mean arterial pressure (MAP), body weight and height at each visit.

### Statistical Analysis

For descriptive purposes, continuous data are shown as mean ± standard deviation (SD) and categorical data are presented as frequencies and percentages (%). At baseline, Student’s *t*-test or the Mann-Whitney *U*-test (in case of a non-normally distributed data) was used to compare the normal and high-risk pregnancies for continuous variables, and a Chi-square test or Fisher’s Exact test for categorical variables.

The following three capillaroscopic parameters, calculated for each patient at each trimester, are used in the statistical models: (1) Cde is the average value of the (at maximum) 16 counts (4 fingers per hand, 2 evaluations per finger. (2) Capillary diameter. A patient’s value is obtained by first averaging the diameters of the (at max) four different fingers of each hand, then the average of these four values per hand and finally the average of the two hands. (3) Total capillary bed is a calculation whereby the average of eight fingers after summation of each digit’s average diameter is multiplied by its average density.

For each capillaroscopic variable, a linear mixed model for repeated measures was employed to compare the evolution of the outcome over time for the two groups per trimester. Trimester, group (low risk vs. high risk) and their interaction were included as fixed explanatory effects in the model with the capillaroscopic variable as dependent variables. A non-linear evolution was allowed by including the trimester as a categorical variable. The linear mixed model also included a random patient effect to take into account the correlation between the capillaroscopic measurements over the different trimesters of the same patient. Based on this model, changes within a group and differences between the groups at the three trimesters were investigated.

MAP and the capillaroscopic variables are associated by means of a linear mixed model. The measurements from all trimesters are used and models with MAP as dependent variable, the capillaroscopic variable as independent variable, the group and their interaction were fitted. Again, a random patient intercept was included. From this model, the change in MAP for a unit change in the capillaroscopic variable is estimated for each group, i.e., the regression slope.

No correction of multiple testing was used. All statistical analyses were performed in SAS v9.4.

## Results

Patient characteristics are depicted in Table 1. The differences between the high risk group vs. low risk group were found in age, parity, BMI, gestational age at birth, birth weight (but not percentile), MAP and medication use. Patients and assessment distributions per trimester are depicted in Table 2. In the low risk group we had 68, 35, and 28 women in each trimester, respectively. Thirty eight women had measurements in first and second trimester. In the high risk group we had 30, 47, and 62 women in each trimester, respectively. Six women had measurements in first and second trimester, 14 women in second and third trimester, 15 women in first and third trimester and two women in all three trimesters.

**TABLE 2:** Distribution of patients and assessments.

Low risk			High risk		
1st trimester	2nd trimester	3rd trimester	1st trimester	2nd trimester	3rd trimester
68	35	28	30	47	62
38			6		
				14	
			——————————————————>15		
			2		
169 women in total			176 women in total (142 with normal outcome,		
207 measurements in total			34 with pre-eclampsia) 215 measurements in total		

### Capillary Density and Diameter

Table 3 shows the trimestral mean and 95% confidence intervals for CDe, CDi, and CB in and between the three groups.

**TABLE 3:** Results.

Results		Trimester 1	Trimester 2	Trimester 3	Trimester 1 → 2	Trimester 2 → 3	Trimester 1 → 3
**Capillary density**	**Low risk group—normal outcome (= A)**	7.31 [7.16; 7.46]	7.38 [7.20; 7.56]	7.01 [6.7; 7.33]	*p* = 0.47202	***p* = 0.04552**	*p* = 0.09430
	**High risk group—normal outcome (= B)**	7.64 [7.41; 7.86]	7.48 [7.27; 7.69]	7.39 [7.20; 7.57]	*p* = 0.28476	*p* = 0.46680	*p* = 0.05786
	**High risk group—pre-eclampsia (= C)**	7.49 [6.85; 8.13]	7.58 [7.15; 8.01]	7.13 [6.80; 7.46]	*p* = 0.80606	*p* = 0.07041	*p* = 0.29217
	**B vs. A**	***p* = 0.01823**	*p* = 0.48037	***p* = 0.04488**			
	**C vs. A**	*p* = 0.59455	*p* = 0.40566	*p* = 0.61220			
	**C vs. B**	*p* = 0.67337	*p* = 0.68045	*p* = 0.18236			
**Capillary diameter**	**Low risk group—normal outcome (= A)**	17.47 [16.89; 18.04]	16.54 [15.86; 17.22]	17.27 [16.08; 18.45]	***p* = 0.01773**	*p* = 0.29655	*p* = 0.76529
	**High risk group—normal outcome (= B)**	17.96 [17.12; 18.79]	17.24 [16.43; 18.04]	17.54 [16.83; 18.24]	*p* = 0.20303	*p* = 0.55957	*p* = 0.40655
	**High risk group—pre-eclampsia (= C)**	19.61 [17.14; 22.06]	15.90 [14.19; 17.61]	16.68 [15.43; 17.93]	***p* = 0.01097**	*p* = 0.42563	***p* = 0.02803**
	**B vs. A**	*p* = 0.34392	*p* = 0.19410	*p* = 0.70031			
	**C vs. A**	*p* = 0.09724	*p* = 0.49330	*p* = 0.50403			
	**C vs. B**	*p* = 0.21371	*p* = 0.16388	*p* = 0.24267			
**Capillary bed**	**Low risk group—normal outcome (= A)**	127.12 [122.6; 131.65]	122.22 [116.88; 127.57]	120.59 [111.37; 129.82]	*p* = 0.11171	*p* = 0.76384	*p* = 0.21216
	**High risk group—normal outcome (= B)**	135.17 [128.6; 141.75]	126.12 [119.8; 132.45]	128.50 [122.95; 134.06]	***p* = 0.04196**	*p* = 0.55298	*p* = 0.09642
	**High risk group—pre-eclampsia (= C)**	146.01 [126.63; 165; 38]	123.53 [110.11; 136.94]	119.79 [110; 129.57]	***p* = 0.04998**	*p* = 0.62926	***p* = 0.01272**
	**B vs. A**	***p* = 0.04801**	*p* = 0.35505	*p* = 0.14936			
	**C vs. A**	*p* = 0.06278	*p* = 0.85932	*p* = 0.90620			
	**C vs. B**	*p* = 0.29834	*p* = 0.73075	*p* = 0.12847			

*This table shows the results per parameter for each trimester and per risk group the mean value and the 95% confidence interval. Significance levels are specified by p-value.*

*Bold values are the significant ones.*

Intertrimestral trends are similar, however, most pronounced in the low risk group where, CDe decreases from second to third trimester and CDi decreases from first to second trimester. We observed significant higher CDe in first and third trimester in women with a high risk cardiovascular profile and normal pregnancy outcome.

### Capillary Bed

Women with high risk cardiovascular profile and normal pregnancy outcome had a significantly greater CB in the first trimester. In women who developed pre-eclampsia, we could observe a significant decrease of CB in the third trimester as compared to previous trimesters of pregnancy, but no differences along trimesters as compared to the other groups.

### Association Between Parameters

An overview of MAP and the difference between groups is shown in Table 4.

**TABLE 4:** Overview of MAP in different groups and per trimester.

		Trimester 1	Trimester 2	Trimester 3	Trimester 1 → 2	Trimester 2 → 3	Trimester 1 → 3
**MAP**	**Low risk group—normal outcome (= A)**	92.38 [90.39; 94.36]	91.82 [89.53; 94.11]	87.85 [83.79; 91.91]	*p* = 0.6659	*p* = 0.0943	*p* = 0.0495
	**High risk group—normal outcome (= B)**	96 [93.22; 98.78]	93.38 [90.78; 95.98]	95.88 [93.53; 98.24]	*p* = 0.1506	*p* = 0.1288	*p* = 0.9428
	**High risk group—pre-eclampsia (= C)**	99.34 [91.05; 107.63]	102.29 [96.71; 107.86]	108.71 [104.55; 112.86]	*p* = 0.5404	***p* = 0.0460**	***p* = 0.0353**
	**B vs. A**	***p* = 0.0374**	*p* = 0.3772	***p* = 0.0008**			
	**C vs. A**	*p* = 0.1086	***p* = 0.0007**	***p* = 0.0000**			
	**C vs. B**	*p* = 0.4526	***p* = 0.0047**	***p* = 0.0000**			

*Bold values are the significant ones.*

Table 5 shows the association between NVC measurements and mean arterial pressure in women with pre-eclampsia. There is a statistical significant correlation between MAP and CDe. We could not observe clinically significant associations in the other groups between MAP, BMI, age, birth weight percentile and CDe/CDi.

**TABLE 5:** Overview of MAP in different groups and per trimester.

	Dependent variable	Independent variable	Estimate	*P*-value
**High risk group—pre-eclampsia**	**Mean arterial pressure**	**Capillary density**	-5.7230	**0.0022**
	**Mean arterial pressure**	**Capillary diameter**	-0.1951	0.7458

*This table depicts the association between CDe and CDi as dependent and independent variables and other variables. Based on the independent variables, we tried to predict the dependent variables. The models assumes a linear association between dependent and independent variables. The strength of association is reflected in the slope (estimate). The estimate depicts the change in dependent variable by increase of 1 unit of the independent variable. The P-value depicts whether there is a significant association or not.*

*Bold values are the significant ones.*

#### Intra-Rater Variability

We did not perform an interrater variability measurement, since in earlier research this is well documented and NVC is found to be a reproducible technique in measurement of CDi and CDe. Although, we calculated an intra-rater variability by repeating the measurements of twenty randomly chosen patients in our cohort. We hereby report the results of intraclass correlation coefficient (ICC), (see Table 6).

**TABLE 6:** Intraclass correlation coefficient analysis (ICC).

	CDe	CDi
Mean value per patient	0.99	0.98

**FIGURE 3:**
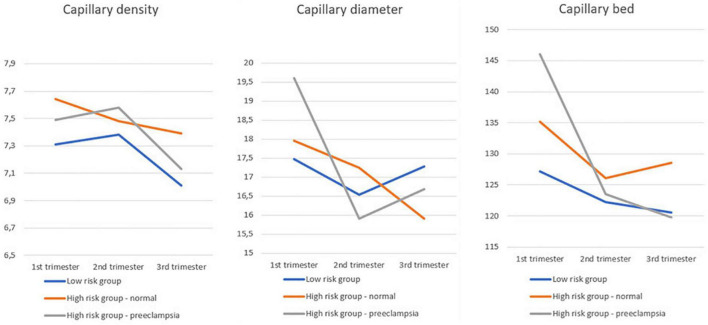
Graphical overview of the mean values of CDe, CDi, and CB, as shown in Table 3.

Figure 3 is a graphical overview of the mean values of CDe, CDi and CB, as shown in Table 3. Per trimester and per risk group the mean values are given, which indicates the global evolution throughout pregnancy.

**FIGURE 4:**
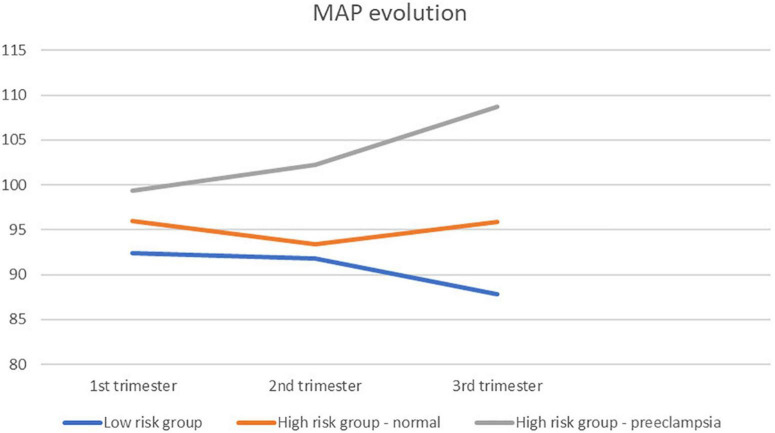
Graphical overview of the mean values of MAP as shown in Table 5.

Figure 4 is a graphical overview of the mean values of MAP as shown in Table 4. Per trimester and per risk group the mean values are given, which indicates the global evolution throughout pregnancy.

## Discussion

### Main Findings

1. Women with a high risk cardiovascular profile show higher CDe, regardless of pregnancy outcome. In women with high risk cardiovascular profile and with normal pregnancy outcome, the higher CDe is higher in first and third trimester as compared with women with low cardiovascular risk.
2. CDi drops in both the low risk and the group of women with preeclampsia from the first to the second trimester. In the low risk group, however, the difference is no longer observed in the third trimester, as is the case in the preeclampsia group.
3. CB, as a composite of CDe and CDi is stable during pregnancy in women with low risk cardiovascular profile. In women with high risk cardiovascular profile, CB drops from the first to the second trimester, regardless of pregnancy outcome. Only in women with pre-eclampsia, the CB is lower in the third trimester as compared to the 1st trimester.
4. There is an inverse correlation between CDe and MAP in women with high cardiovascular risk and pre-eclampsia.
5. In this study we were able to show the feasibility and potential of microvascular assessment using NVC in pregnant women. The easy accessibility makes it an attractive technique to use, since there is no need to a special environment to perform the examination. It is done by a handheld, USB coupled device, which can be used even bedside.

### Interpretation

Microcirculatory assessment using NVC has previously been described in women with pregnancy induced hypertension (3, 7).

More recent research also investigated the role of NVC in normal pregnancy course (15). Assessment of the maternal microvascular system can offer insights in the maternal global cardiovascular and placental function and in the pathophysiological processes of hypertensive complications of pregnancy (16).

CDi seems to compensate to findings, seen in CDe in patients with low cardiovascular risk. CDi drops from the first to the second trimester, but in the third trimester there is no longer a significant lower CDi as compared to the first trimester. Meanwhile, CDe drops significant from the second to the third trimester. We can speculate that by lowering CDe, a compensatory vasodilatation occurs (CDi rises). This is different in patients with a high cardiovascular risk with evolution toward pre-eclampsia, there we do observe a lower CDi in the third trimester as compared to the first trimester, reflective of a vasoconstrictive status. CDe shows, however, no significant drop. Further research in conjunction with macrocirculatory assessment is needed to clarify these findings.

Our data on CDe are in line with earlier published research. Capillary rarefaction (defined as the reduction in the number of capillaries per visual field), described in normal pregnancy, is consistent with the significant drop in CDe in our study in the group of women with low cardiovascular risk (17). In earlier animal models, this was shown in multiple microvascular beds (18, 19). CDe shows no difference between the high risk group with preeclampsia and the other groups. This finding is also in line with earlier research in skin capillaroscopy (20). Our data show differences in CDe but in a selected group of women with high cardiovascular risk and normal pregnancy outcome. They have higher CDe in first and third trimester of pregnancy, which is surprising, since we hypothesized it would be similar regardless of cardiovascular risk. Whether this finding is linked to the eventual normal gestational outcome is an interesting topic for further research. Speculative is that the lack of rarefaction (higher CDe) is possibly protective against a raise in blood pressure and evolution toward pre-eclampsia, which makes that women with a high cardiovascular risk and a higher CDe (in comparison to women with normal cardiovascular risk) are more likely to have a normal pregnancy outcome. Our groups are too small to make distinction between early and late pre-eclampsia, which could be of interest. In further analysis, when also taking into account macrocirculatory parameters and/or serum biomarkers that reflect vascular remodeling. Of course, to further clarify this finding, we have to look at the macrocirculatory parameters in both groups, which is part of our future research. But, in this way, capillaroscopy can be a potentially new tool in addition to the other assessments, to make a distinction between women with high risk pregnancy who will have low or high risk to develop pre-eclampsia.

Although further research is needed, the process of capillary rarefaction seems to precede the onset of hypertension, suggesting that it is a primary phenomenon, rather than secondary (21). In high risk patients, capillary rarefaction can be a positive predictor for the development of pre-eclampsia, which could further increase sensitivity and specificity of uterine artery Doppler pulsatility index for the development of PE (4). In our preeclampsia group we confirmed the finding of capillary rarefaction, as CDe and MAP are inversely related with high statistical significance.

From the physiological point of view, microcirculatory function relates to both arterial and venous vascular activity. Arterial vasoconstriction reduces the capillary perfusion, whereas the opposite is true for venous congestion. Our data show that in early pregnancy of PE pregnancies, an expanded capillary bed is present, evolving to normal during the course of pregnancy in association with a rise of blood pressure. One conclusion might be that in these women, venous congestion is already present in the early stages of pregnancy, reducing to normal levels with increasing arteriolar constriction and blood pressure. The hypothesis of gestation induced hypertension as a protective mechanism against microcirculatory congestion is very interesting and invites for further assessment in future research (22).

CDe and CDi measurement by NVC, are useful and feasible. They add information on the pathophysiology of hypertensive pregnancy disorders. As a standalone examination, they cannot discriminate, based on the current data, but together with macrocirculatory measurements, a more detailed profile of the total hemodynamic status in pregnancy can be generated. Based on our current study and on earlier published data, it is not yet possible to really define cut-off values. We can measure and see a trend, but variation throughout pregnancy is too small to make distinction between high and/or low and/or normal. Values are likely to be seen in relation to other measurements, but of course at this stage this is rather speculative. For further research, as microcirculation is influenced by pre- and postcapillary factors, it would be of uttermost interest to couple macrovascular observations to microvascular changes as they form a continuum and integral part of the cardiovascular system, to search for correlations, that can explain the microcirculatory observations. Despite normal pregnancy outcome, we observed differences based on cardiovascular risk profiles, which could be of value for early detection of cardiovascular events later on in life. Future research should also focus on microcirculatory changes in women with and without hypertensive and/or uteroplacental complications in pregnancy, taking the cardiovascular risk profile into account.

### Strengths and Limitations

Our strength is the higher number of patients included. Most research on microcirculation in pregnancy is performed in small populations. We described the findings throughout pregnancy in both a large low risk, high risk with normal outcome and high risk who developed PE. In this way, it is reassuring that our results in a larger population are in line with earlier published research in smaller populations.

Limitations are the lack of consistently, consecutive measurements in each subject. The population is a mix of longitudinal and cross-sectional measurements for each group, in each trimester. In the high risk group, this is due to the fact that patients were mostly seen once, as a tertiary advice for the peripheral colleague gynecologists. Patients are not routinely seen twice in our center. In the normal risk group, we did had the intention to see patients four times (during pregnancy and once post-partum) but due to COVID-19 pandemic, only necessary visits were allowed. Second limitation is that we don’t have equal number of patients in every trimester. This last limitations is somewhat overcome by our higher number of patients in total.

## Conclusion

With our observational study, we could confirm that microcirculation is altered during the course of pregnancy and that there is a difference in patients with low and with high cardiovascular risk profile, as well as in patients with high risk profile who do and do not develop preeclampsia. As capillary rarefaction seems to precede the onset of PE, capillaroscopy could be of diagnostic value in predicting pre-eclampsia in high risk populations.

## Data Availability Statement

The raw data supporting the conclusions of this article will be made available by the authors, without undue reservation.

## Ethics Statement

The studies involving human participants were reviewed and approved by the Ethical Committee ZOL Genk. The patients/participants provided their written informed consent to participate in this study.

## Author Contributions

KT and MD gathered data and analyzed the data. KT wrote the article. MD, JC, and WG scrutinized the article to further optimization by providing insight and suggestions. JC and WG were guiding KT in his Ph.D. trajectory. All authors contributed to the article and approved the submitted version.

## Conflict of Interest

The authors declare that the research was conducted in the absence of any commercial or financial relationships that could be construed as a potential conflict of interest.

## Publisher’s Note

All claims expressed in this article are solely those of the authors and do not necessarily represent those of their affiliated organizations, or those of the publisher, the editors and the reviewers. Any product that may be evaluated in this article, or claim that may be made by its manufacturer, is not guaranteed or endorsed by the publisher.
