# Corrigendum: Nailfold video capillaroscopy in pregnant women with and without cardiovascular risk factors

**DOI:** 10.3389/fmed.2022.1049459

**Published:** 2023-05-10

**Authors:** Kristof Thevissen, Merve Demir, Jerome Cornette, Wilfried Gyselaers

**Affiliations:** ^1^Department of Rheumatology, Ziekenhuis Oost-Limburg (ZOL), Genk, Belgium; ^2^Faculty of Medicine and Life Sciences, Hasselt University, Diepenbeek, Belgium; ^3^Department of Obstetrics and Fetal Medicine, Erasmus Medical Center, Rotterdam, Netherlands; ^4^Department of Physiology, Hasselt University, Diepenbeek, Belgium; ^5^Department of Gynecology, Ziekenhuis Oost-Limburg (ZOL), Genk, Belgium

**Keywords:** nailfold video capillaroscopy, microcirculation, pregnancy, pre-eclampsia, hypertension

In the published article, there was an error with the author names, the surnames and first names were in the wrong order. The correct author details appear below:

Author list: Kristof Thevissen, Merve Demir, Jerome Cornette, Wilfried Gyselaers

The authors apologize for this error and state that this does not change the scientific conclusions of the article in any way. The original article has been updated.

